# Community-driven updates for comprehensive long-read metagenomics and enhanced binning in nf-core/mag v5

**DOI:** 10.1093/bioinformatics/btag628

**Published:** 2026-08-25

**Authors:** Diego Alvarez Saravia, Adam Rosenbaum, Daniel Straub, Jim Downie, Maxime Borry, Greg Fedewa, Alexander Hübner, Daniel Lundin, Jeferyd Yepes-García, James McDonald, Sven Nahnsen, Linda Köhn, Roberto Uribe-Paredes, Marcelo A Navarrete, Christina Warinner, nf-core community, James A Fellows Yates

**Affiliations:** Department of Computer Engineering, Universidad de Magallanes, Punta Arenas, Magallanes, 6200000, Chile; School of Medicine, Universidad de Magallanes, Punta Arenas, Magallanes, 6200000, Chile; Department of Medical Biosciences, Umeå University, Umeå, Västerbotten, 90187, Sweden; Quantitative Biology Center (QBiC), University of Tübingen, Tübingen, Baden-Württemberg, 72076, Germany; M3 Research Center, University of Tübingen, Tübingen, Baden-Württemberg, 72076, Germany; Tree of Life Programme, Wellcome Sanger Institute, Wellcome Genome Campus, Hinxton, Cambridgeshire, CB10 1SA, United Kingdom; Molecular Ecology and Evolution, Bangor University, Environment Centre Wales, Bangor, Gwynedd, LL57 2DG, United Kingdom; Microbiome Sciences Group, Department of Archaeogenetics, Max Planck Institute for Evolutionary Anthropology, Leipzig, Saxony, 04103, Germany; Independent Researcher, Pasadena, CA, United States; Microbiome Sciences Group, Department of Archaeogenetics, Max Planck Institute for Evolutionary Anthropology, Leipzig, Saxony, 04103, Germany; Associated Research Group Archaeogenetics, Leibniz Institute for Natural Product Research and Infection Biology, Jena, Thuringia, 07745, Germany; EEMiS, Linnaeus University, Kalmar, 39182, Sweden; Department of Biology, University of Fribourg, Fribourg, Fribourg, 1700, Switzerland; BUGFri, Swiss Institute of Bioinformatics, Amphipole, Quartier UNIL-Sorge, Lausanne, Vaud, 1015, Switzerland; Molecular Ecology and Evolution, Bangor University, Environment Centre Wales, Bangor, Gwynedd, LL57 2DG, United Kingdom; Birmingham Institute of Forest Research, Institute of Microbiology and Infection, School of Biosciences, University of Birmingham, Birmingham, B15 2TT, United Kingdom; Quantitative Biology Center (QBiC), University of Tübingen, Tübingen, Baden-Württemberg, 72076, Germany; M3 Research Center, University of Tübingen, Tübingen, Baden-Württemberg, 72076, Germany; FBI/IBMI-Biomedical Data Science, University of Tübingen, Tübingen, Baden-Württemberg, 72076, Germany; Department of Medical Biosciences, Umeå University, Umeå, Västerbotten, 90187, Sweden; Department of Computer Engineering, Universidad de Magallanes, Punta Arenas, Magallanes, 6200000, Chile; School of Medicine, Universidad de Magallanes, Punta Arenas, Magallanes, 6200000, Chile; Microbiome Sciences Group, Department of Archaeogenetics, Max Planck Institute for Evolutionary Anthropology, Leipzig, Saxony, 04103, Germany; Associated Research Group Archaeogenetics, Leibniz Institute for Natural Product Research and Infection Biology, Jena, Thuringia, 07745, Germany; Department of Anthropology, Harvard University, Cambridge, MA 02138, United States; nf-core, Solna, Sweden; Microbiome Sciences Group, Department of Archaeogenetics, Max Planck Institute for Evolutionary Anthropology, Leipzig, Saxony, 04103, Germany; Associated Research Group Archaeogenetics, Leibniz Institute for Natural Product Research and Infection Biology, Jena, Thuringia, 07745, Germany; Department of Palaeobiotechnology, Leibniz Institute for Natural Product Research and Infection Biology, Jena, Thuringia, 07745, Germany

## Abstract

**Summary:**

nf-core/mag is a reproducible Nextflow pipeline for best-practice metagenomic *de novo* assembly and binning within the nf-core framework. Here we present a major update that adds support for long-read-only assembly and bin refinement, includes five new binning tools, expands taxonomic classification to viruses and eukaryotes, and improves bin quality evaluation with new tools and latest databases. Through sustained community-driven development spanning seven years and four primary curator teams, nf-core/mag remains actively developed as an open source workflow for metagenomic analysis, benefiting from contributions from across the broader metagenomics, nf-core, and Nextflow ecosystem.

**Availability and implementation:**

The source code of nf-core/mag v5 is available on GitHub (https://github.com/nf-core/mag) under the open source MIT license, with v5.5.0 source code archived on Zenodo (https://zenodo.org/records/21735731). Documentation is viewable on the nf-core website (https://nf-co.re/mag).

## 1 Introduction

Reference-free recovery of microbial genome sequences through metagenomic *de novo* assembly has revolutionized scientists’ ability to study difficult-to-isolate taxa and complex microbial ecosystems (Liu *et al.* 2025).

The generation of Metagenome-Assembled Genomes (MAGs) typically involves four major stages (Liu *et al.* 2025): read pre-processing, assembly of reads to build contigs and/or scaffolds, algorithmic ‘binning’ or clustering of related contigs putatively deriving from the same genome, and finally functional and taxonomic annotation and quality control evaluation of MAGs against metrics such as the Minimum Information about a Metagenome-Assembled Genome (MIMAG) criteria (Bowers *et al.* 2017).

Typically, metagenomic *de novo* assembly has been performed on relatively deep-sequenced short but highly accurate reads, such as from Illumina sequencers. However, large improvements in the error rates of long-read sequencing platforms have resulted in increasing popularity in the use of long-read datasets for assembly. Long reads can span across difficult-to-assemble regions (including key marker genes such as the 16S rRNA gene), improve strain-level taxonomic identification, and generally result in less fragmented assemblies (Agustinho *et al.* 2024).

**Figure 1:**
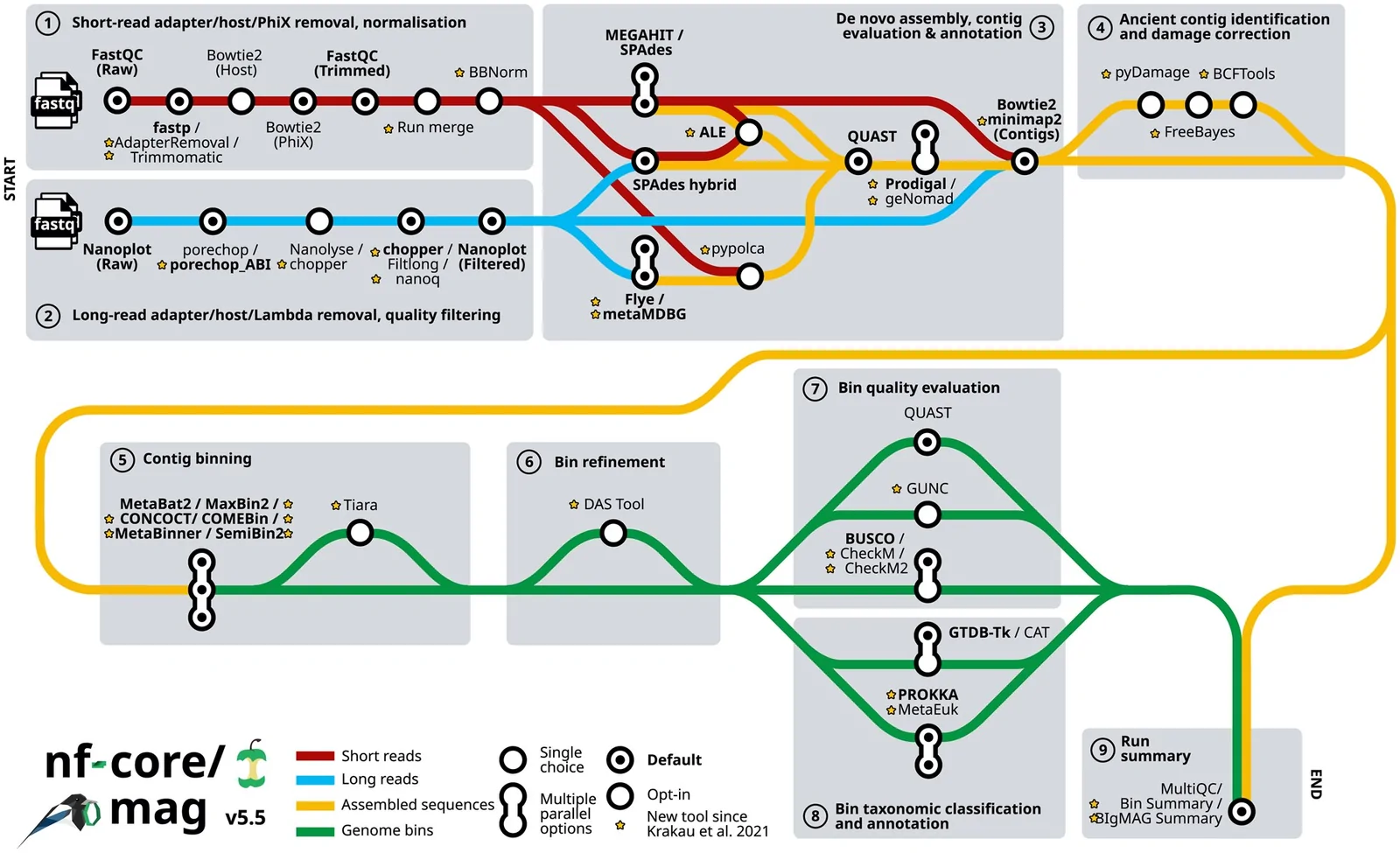
Schematic diagram of the nf-core/mag pipeline as of v5.5. The pipeline consists of two separate subworkflows for preparing short-read and long-read sequencing reads respectively. Reads are then assembled either in single or hybrid mode as per user choice. Contigs are binned with a range of binners and bins can be optionally refined using DAS Tool. Resulting bins are annotated functionally and taxonomically, and quality control metrics generated. Overall pipeline run statistics are summarized with MultiQC and bin results in a dedicated results table and a BIgMAG compatible file.

We have previously described the pipeline nf-core/mag (Krakau *et al.* 2022), an open-source Nextflow best-practice pipeline for metagenome assembly, binning, and taxonomic classification, that supported short-read co-assembly as well as combined short- and long-read hybrid assembly with two assemblers and a single binner. Here we provide updates on the nf-core/mag project, with new functionality including standalone long read assembly with two additional assemblers, new binning and quality control tools, and support for bin refinement (Fig. 1).

## 2 Expanded capabilities overview

### 2.1 Long-read only support

In the latest major release of nf-core/mag, the pipeline adds support for independent metagenome assembly for long-read sequencing data. Whereas earlier releases of the pipeline allowed long-read data as input, it was used exclusively for hybrid assembly with metaSPAdes (Nurk *et al.* 2017), which uses long reads to help close gaps between short-read assembled contigs.

Users are now required to specify the sequencing platform as metadata in the input sample sheet, allowing the pipeline to automatically adjust optimized parameters for each platform.

Oxford Nanopore raw read pre-processing now includes trimming and filtering with either porechop-ABI (Bonenfant *et al.* 2023), chopper (De Coster and Rademakers 2023), or nanoq (Steinig and Coin 2022), followed by an optional host-removal step. NanoPlot (De Coster and Rademakers 2023) is applied to both raw and processed reads.

At the assembly stage, the pipeline introduces two dedicated long-read metagenomic assemblers. (meta)Flye (Kolmogorov *et al.* 2020) is a mature, well-established assembler that builds and resolves a repeat graph to handle non-uniform coverage and complex inter-genomic repeats. metaMDBG (Benoit *et al.* 2024) is a more recent alternative that assembles a de Bruijn graph in minimizer space with a multi-k iterative strategy and abundance-based filtering, which is generally very efficient and has recently been updated to improve support for noisy reads (Benoit *et al.* 2026). Both enable the recovery of high-quality MAGs from complex microbial communities using long reads alone.

Downstream of assembly, the workflow follows the same binning and refinement steps as short-read or hybrid assemblies, ensuring consistent processing of MAGs regardless of input data type.

### 2.2 Binning improvements

The options for contig binning have also been expanded, with nf-core/mag now offering the simultaneous execution of MetaBAT2 (Kang *et al.* 2019), MaxBin2 (Wu *et al.* 2016), CONCOCT (Alneberg *et al.* 2014), COMEBin (Wang *et al.* 2024), MetaBinner (Wang *et al.* 2023), and SemiBin2 (Pan *et al.* 2023). These binners employ diverse approaches for grouping contigs: MaxBin2 uses an expectation-maximization algorithm, CONCOCT utilizes coverage across multiple samples and tetranucleotide frequencies with Gaussian mixture models, COMEBin applies contrastive multi-view representation learning to generate embeddings from sequence coverage and k-mer distributions, MetaBinner is an ensemble method that uses single-copy gene information to guide k-means clustering, and SemiBin2 employs self-supervised deep learning with siamese neural networks.

After running multiple binners, metagenomic researchers are increasingly ‘refining’ the set of bins to remove duplicate or contaminated bins and select the highest quality version (Song and Thomas 2017). nf-core/mag now integrates DAS Tool (Sieber *et al.* 2018), which can be enabled when multiple binners are run. DAS Tool evaluates redundancy and overlap of bins, selecting the best representative for each genomic cluster based on single-copy gene completeness and contamination metrics.

### 2.3 Broader taxonomic support

Contig taxonomic classification capabilities have been expanded to include viral and eukaryotic sequences, providing a comprehensive description of microbial communities.

For viral identification and classification, the pipeline now includes geNomad (Camargo *et al.* 2024). This tool uses both gene-based classification and deep learning models to detect viral sequences within metagenomic assemblies and bins.

Inspired by VEBA (Espinoza and Dupont 2022), nf-core/mag now also includes a domain classification subroutine to classify bins to the domain level. First, contigs are classified with Tiara (Karlicki *et al.* 2022), which uses deep learning to quickly classify sequences into taxonomic categories (Archaea, Bacteria, Eukaryota, organelles, or unknown). The contig-level classifications for each bin are then aggregated and averaged to produce an overall domain-level classification for each bin. These classifications can be used to filter inputs downstream to different processes, e.g. excluding eukaryotic bins from prokaryote-specific tools such as GTDB-Tk (Chaumeil *et al.* 2022) or Prokka (Seemann 2014).

When domain classification is enabled, eukaryotic bins are instead annotated with MetaEuk (Levy Karin *et al.* 2020), providing fast and sensitive gene prediction on their contigs.

### 2.4 Bin quality evaluation

Bin quality assessment has been improved with the addition of new tools, providing users with multiple metrics for MAG validation.

Both CheckM (Parks *et al.* 2015) and the more recent CheckM2 (Chklovski *et al.* 2023) have been integrated, which estimate genome completeness and contamination based on lineage-specific gene markers and machine learning models trained on extensive genomic databases, respectively.

In addition, BUSCO (Manni *et al.* 2021) has been upgraded to its latest version (v6), expanding its reference lineage datasets and improving the assessment accuracy for multiple taxonomic groups.

To support bin quality exploration through a visual and interactive strategy, nf-core/mag now features the generation of an input file for BIgMAG (Yepes-García and Falquet 2024). This file is obtained by concatenating the outputs from BUSCO, CheckM2, GUNC (Orakov *et al.* 2021), QUAST (Mikheenko *et al.* 2016), and GTDB-Tk. As a result, it offers a comprehensive overview of bin quality and taxonomy to extract sample-focused preliminary insights and comparisons.

### 2.5 Additional notable updates

Assembly polishing capabilities have been added by integrating Pypolca (Bouras *et al.* 2024), which corrects errors on long-read assemblies using short-read data.

BBNorm from the BBTools suite (https://jgi.doe.gov/data-and-tools/software-tools/bbtools/) has been added as an optional normalization step for short-read data. This tool reduces coverage variation across samples by normalizing read depth, which can decrease computational requirements for assembly while maintaining assembly quality.

Post-binning taxonomic classification has been enhanced with the option of using the new version of CAT_pack (von Meijenfeldt *et al.* 2019) (v6) to additionally classify unbinned contigs, allowing comprehensive taxonomic profiling of assembled sequences. Both CAT_pack and GTDB-Tk now utilize their latest reference databases for more accurate taxonomic assignments.

One deprecation from the original version of nf-core/mag is the removal of raw read taxonomic classification. This is to maintain a focused scope on metagenome assembly and binning. For this feature, users who wish to stay in the nf-core ecosystem are encouraged to use specialized pipelines such as nf-core/taxprofiler (Stamouli *et al.* 2023).

### 2.6 Community development and longevity

The first release of nf-core/mag was in 2019 (Table 1). This means the current lifespan of seven years of active development has exceeded the typical maintenance period for project-specific scientific software (Malviya-Thakur *et al.* 2025). We attribute this successful longevity of the pipeline to the nf-core community model of development (Ewels *et al.* 2020).

nf-core/mag is not owned or supported by a single group, institution, nor funded by a particular project. Instead, it is maintained by the nf-core community of currently 10 000+ scientists and software engineers (Langer *et al.* 2025). This open research software community concept has enabled the seamless transition across four ‘primary curator’ teams during the lifetime of the pipeline so far (Fig. S1), with developers dropping and picking up the project when their time and career allowed.

The pipeline has also benefited strongly from the centralized nf-core/modules repository of Nextflow command-line tool wrappers. These can be installed in a Nextflow workflow and connected together like ‘building blocks’ to efficiently build pipelines with pre-built components. The nf-core/mag developers have contributed modules now used in other Nextflow metagenomic pipelines, and likewise the developers of these non nf-core pipelines have contributed modules now also used in nf-core/mag (Richardson *et al.* 2023, https://github.com/sanger-tol/metagenomeassembly). This symbiotic relationship has also benefited nf-core/mag by generating good relationships with these other pipeline developers outside of the nf-core ecosystem to the point where the pipeline enjoys a larger pull-request reviewer pool. This in turn is ensuring a higher-quality code base, as well as a larger pool of experts from diverse backgrounds to help guide the development of the pipeline as community needs evolve.

## 3 Computational resource requirements

Computational requirements vary considerably with input size and complexity, experimental design, and available infrastructure. To provide practical guidance, we benchmarked nf-core/mag v5.5.0 on two publicly available metagenomic datasets with matched short- and long-read data from the same samples: the ZymoBIOMICS Fecal Reference (Zymo Research, Irvine, CA, USA) and ten human gut metagenomes from Maghini *et al.* (2025). Taxonomic profiling with sylph (Shaw and Yu 2025) against GTDB R232 (Parks *et al.* 2026) detected 651 species in the Zymo reference and 144 to 403 species (median 319) in the individual gut metagenomes. Short-read depth ranged from 6.9 to 31.7 Gbp per sample and long-read depth from 3.8 to 13.7 Gbp, with read N50 between 3.4 and 13.3 kb.

To bound the upper end of the range, we enabled every assembler and binner and ran all post-binning steps on all bin sets. This exhaustive configuration required approximately 1200 CPU core-hours per sample, with bin classification using CAT adding a further 880 core-hours where enabled (Fig. S2). A representative single-assembler configuration (MEGAHIT with MetaBAT2 and SemiBin2), estimated from the respective subset of tasks, would require fewer than 50 core-hours per sample.

Peak memory is typically determined by GTDB-Tk, for which a maximum of 140 GB should be allocated, followed by CAT and short-read metaSPAdes assembly (about 110–120 GB); MEGAHIT and the long-read assemblers remain below 40 GB (Supplementary Figs. S4 and S5). Computing time is dominated by CAT where enabled, then gene annotation, binning, and bin quality control (Fig. S2). Among binners, COMEBin required by far the most CPU hours, whereas MetaBAT2, MaxBin2, and SemiBin2 required very little (Fig. S4). Requirements scale approximately linearly with sequencing input for most assemblers, whereas binner CPU time grows superlinearly with assembly size (log–log slopes up to 2.3; Supplementary Figs. S6 and S7).

Storage should also be considered, as intermediate results are staged in Nextflow’s work directory, which required a median of 380 GB per sample, but can be cleared once the final outputs are published. Cumulative writes are much larger (median 5.3 TB per sample) but reflect I/O volume rather than capacity (Fig. S3).

A resource-guidance page (https://nf-co.re/mag/5.5.0/docs/usage/resource_guidance) summarizes default configurations and how to tune them. The execution traces, per-sample sequencing depth, read length and assembly size, and the analysis notebooks are archived on Zenodo at https://doi.org/10.5281/zenodo.21809776, together with the compute platform and pipeline configuration used for the runs.

## 4 Conclusion

The development of nf-core/mag will continue into the future, offering efficient, scalable, and reproducible assembly of metagenomes to, and in collaboration with, the scientific community. We aim to continue to add the latest state-of-the-art assemblers and binning tools, by the consensus and requests of the users—particularly as long-read sequencing becomes more prevalent and methods become more advanced. In the near future we wish to provide alternative bin refinement tools, such as Binette (Mainguy and Hoede 2024), add bin dereplication with tools such as dRep (Olm *et al.* 2017), and generate additional quality control (QC) metrics to facilitate easier user-evaluation of their results against the MIMAG criteria. Finally, we wish to work towards better interoperability with upstream and downstream Nextflow pipelines, such as replacing host-removal functionality with the dedicated nf-core/detaxizer pipeline (Seidel *et al.* 2025) and also downstream with the under development nf-core/seqsubmit pipeline (https://nf-co.re/seqsubmit) for automated submission of assemblies and MAGs to databases such as MGnify (Richardson *et al.* 2023). Through continued community-maintenance of nf-core/mag, we aim to keep the pipeline up to date with the latest rapid developments in metagenomic assembly, and provide further efficiency and user-choice in the generation and analysis of MAGs.

**Table 1:** Comparison of a subset of features between the nf-core/mag pipeline and other comparable metagenome assembly pipelines with recent releases (2023 and later): ATLAS (Kieser *et al.* 2020), SqueezeMeta (Tamames and Puente-Sánchez 2018), mmlong2 (Sereika *et al.* 2025), and MAGNETO (Churcheward *et al.* 2022).

	nf-core/mag	ATLAS	SqueezeMeta	mmlong2	MAGNETO
Development span	2019–2026	2017–2024	2018–2026	2023–2025	2023–2026
Code contributors	47	26	3	1	8
Workflow manager	Nextflow	Snakemake	–	Snakemake	Snakemake
Containerization	Conda, Docker, Singularity, …	Conda	Conda	Conda, Singularity	Conda
Read assembly type	short, long, hybrid	short, hybrid	short, long	long	short
Binners available	6	2	3	4	3
Bin refinement	Yes	Yes	Yes	Yes	Yes
Bin dereplication	No	Yes	No	No	Yes
Taxonomic classification	Yes	Yes	Yes	Yes	Yes
Gene prediction	Yes	Yes	No	Yes	Yes

A detailed comparison, with data derived from 2Pipe organization (Yepes-García and Falquet 2026), that includes additional pipelines and factors such as platforms supported, usage metrics, tools encompassed, type of input data (e.g. short reads, long reads, or hybrid), and special features is available (Table S1).

## Supplementary Material

btag628_Supplementary_Data

## Data Availability

The source code of nf-core/mag v5 is available on GitHub (https://github.com/nf-core/mag) under the open source MIT license, with v5.5.0 source code archived on Zenodo (https://zenodo.org/records/21735731). Documentation is viewable on the nf-core website (https://nf-co.re/mag).
