# Supplementary material for: Community-driven updates for comprehensive long-read metagenomics and enhanced binning in nf-core/mag v5
Source: Bioinformatics. 2026 Aug 25;42(9):btag628. doi: 10.1093/bioinformatics/btag628 (PMC13544955; doi:10.1093/bioinformatics/btag628)
Supplement: Supplementary file 1 [file btag628_supplementary_data.zip › mag_v5__one_figure_-supplement.pdf]

Community-driven updates for comprehensive long-read metagenomics and enhanced binning in nf-core/mag v5

Supplementary material

Diego Alvarez Saravia, Adam Rosenbaum, Daniel Straub, Jim Downie, Maxime Borry, Greg Fedewa, Alexander Hübner, Daniel Lundin, Jeferyd Yepes-García, James McDonald, Sven Nahnsen, Linda Köhn, Roberto Uribe-Paredes, Marcelo A. Navarrete, Christina Warinner, nf-core community, and James A. Fellows Yates

Supplementary Table S1. Comparison of features between the nf-core/mag pipeline and other comparable metagenome assembly pipelines with recent releases (2023 and later). Created with data derived from 2Pipe organization.

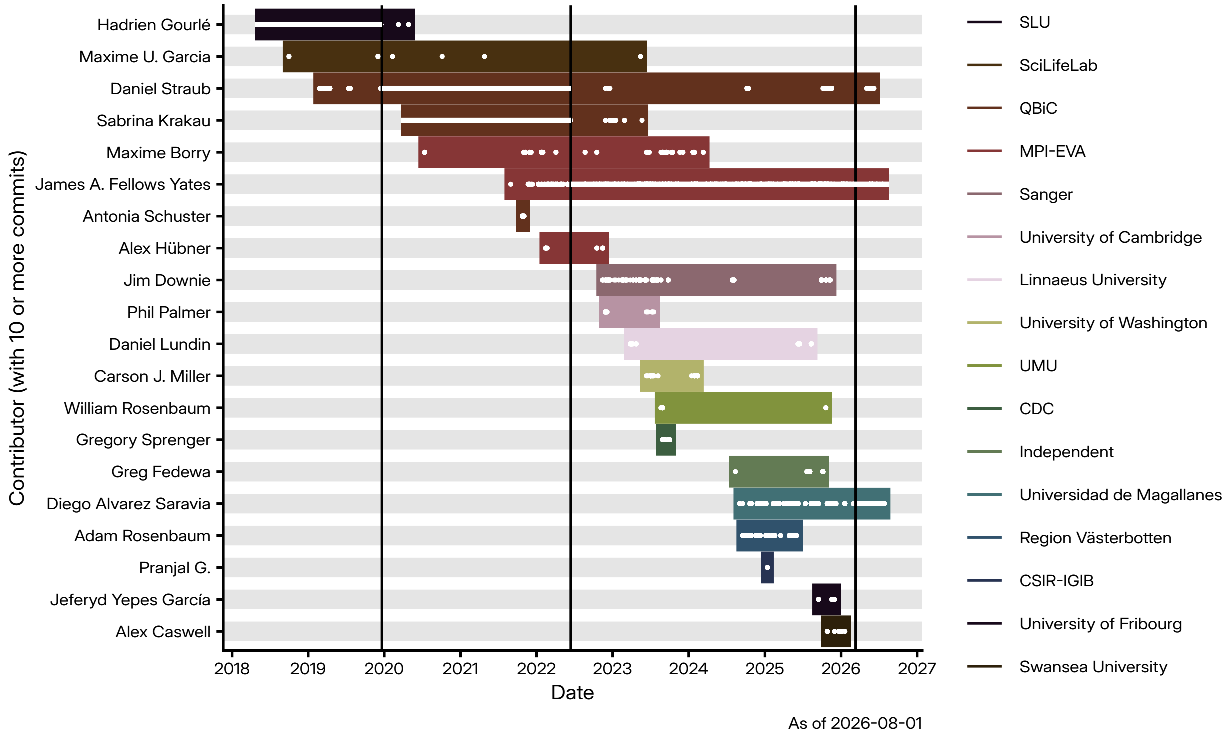

Supplementary Figure S1. Timeline of code contributors to nf-core/mag with more than 10 commits as of August 2026. Coloured bars represent entire span of contribution period, with white dots representing weeks where a commit was made. Black vertical bars represent transition points between different 'primary curators' of the pipeline, each coming from a different organisations. Affiliations represent main affiliation during period of most contributions. Data and code notebook for figure generation archived at: `https://doi.org/10.5281/zenodo.18267864`.

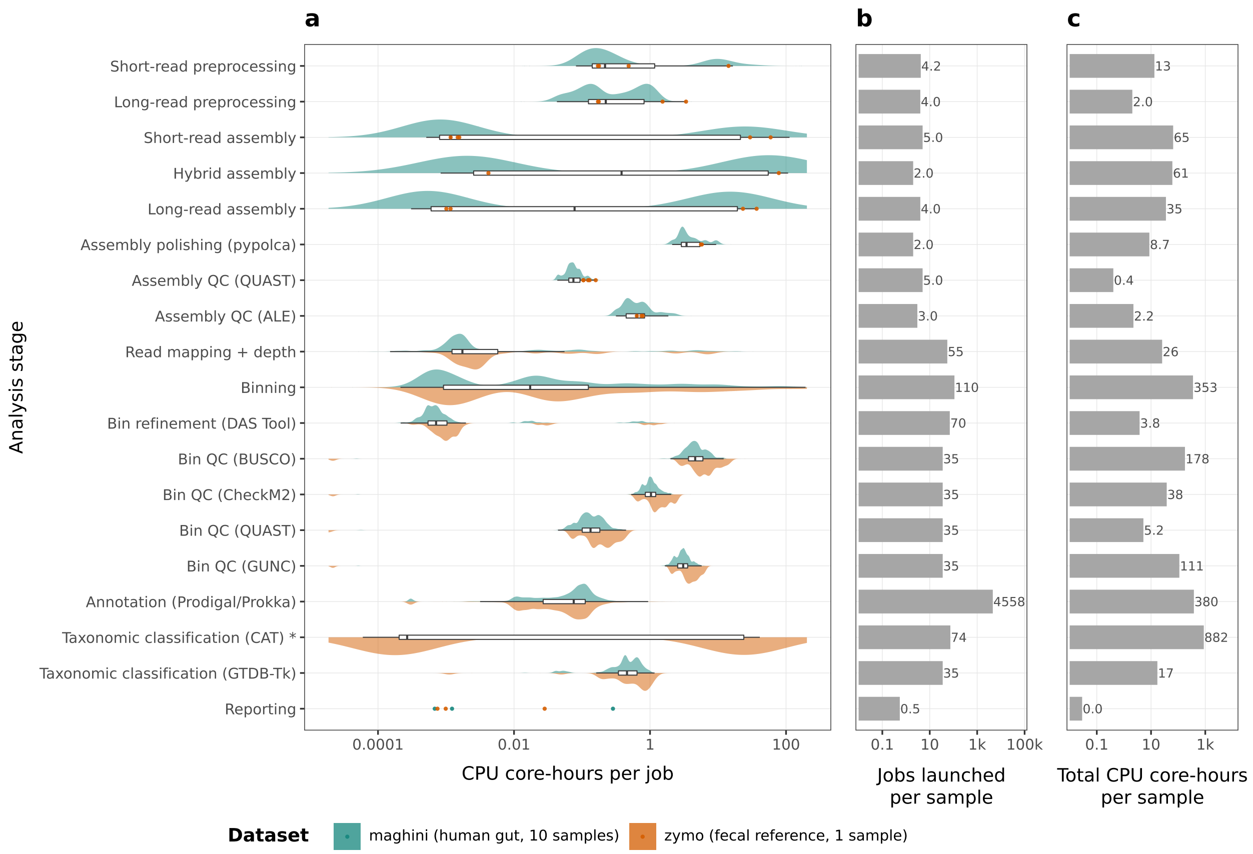

Supplementary Figure S2. Compute use by analysis stage. CPU core-hours per job (a), jobs launched per sample (b), and total CPU core-hours per sample (c). Analysis stages group each main tool with its supporting jobs (for example, bin refinement covers DAS Tool itself plus the renaming and contig-to-bin conversion jobs it requires), so job counts do not necessarily equal the number of tools run. In (a), one kernel density per dataset is shown on either side of each stage, scaled to a common height, over a box-plot pooling both datasets; datasets with too few jobs for a density appear as points. The bars in (b) and (c) pool both datasets by dividing the total by the number of samples. Taxonomic classification (CAT), marked with an asterisk, ran on the ZymoBIOMICS data only, due to its high computational cost.

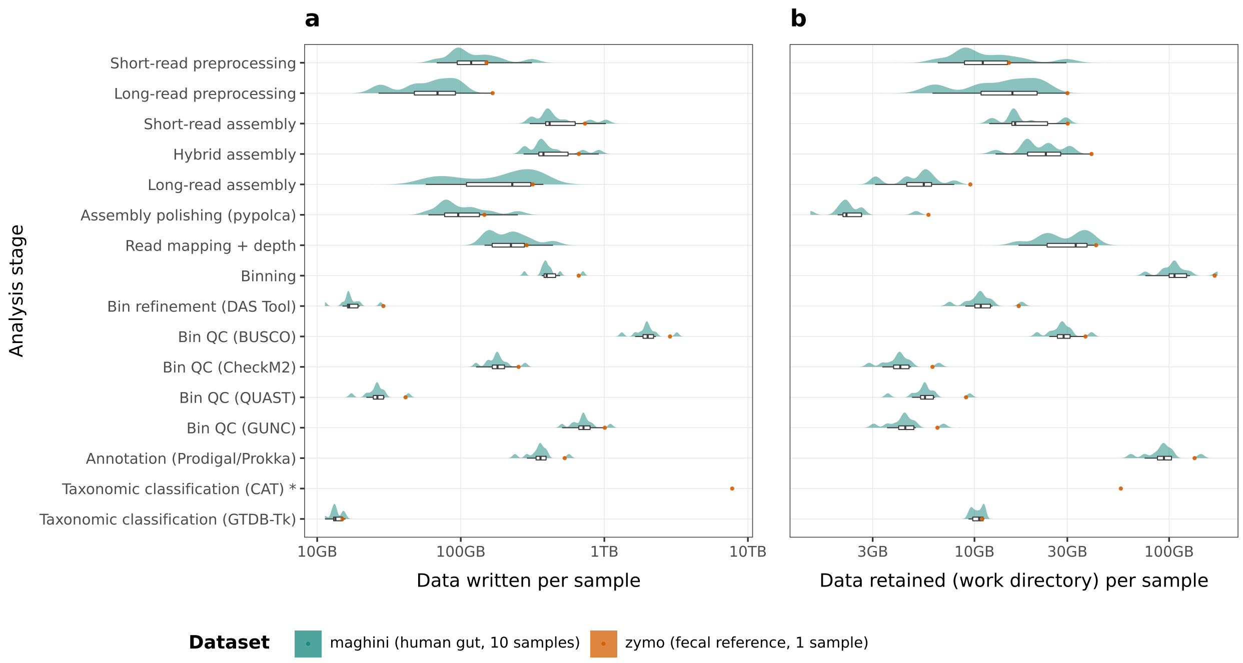

Supplementary Figure S3. Storage per analysis stage. Data written (a) and data retained in the work directory (b) by each analysis stage, expressed per sample. Written data includes temporary files that may be removed before workflow completion. One kernel density scaled to a common height is shown for maghini at each stage, while zymo appears as points, since it has a single observation per stage. The box-plot in the middle pools both datasets. Taxonomic classification (CAT), marked with an asterisk, ran on the ZymoBIOMICS data only, because of its high computational cost. Assembly QC with QUAST and ALE is omitted, since both leave under 10 MB per sample on disk.

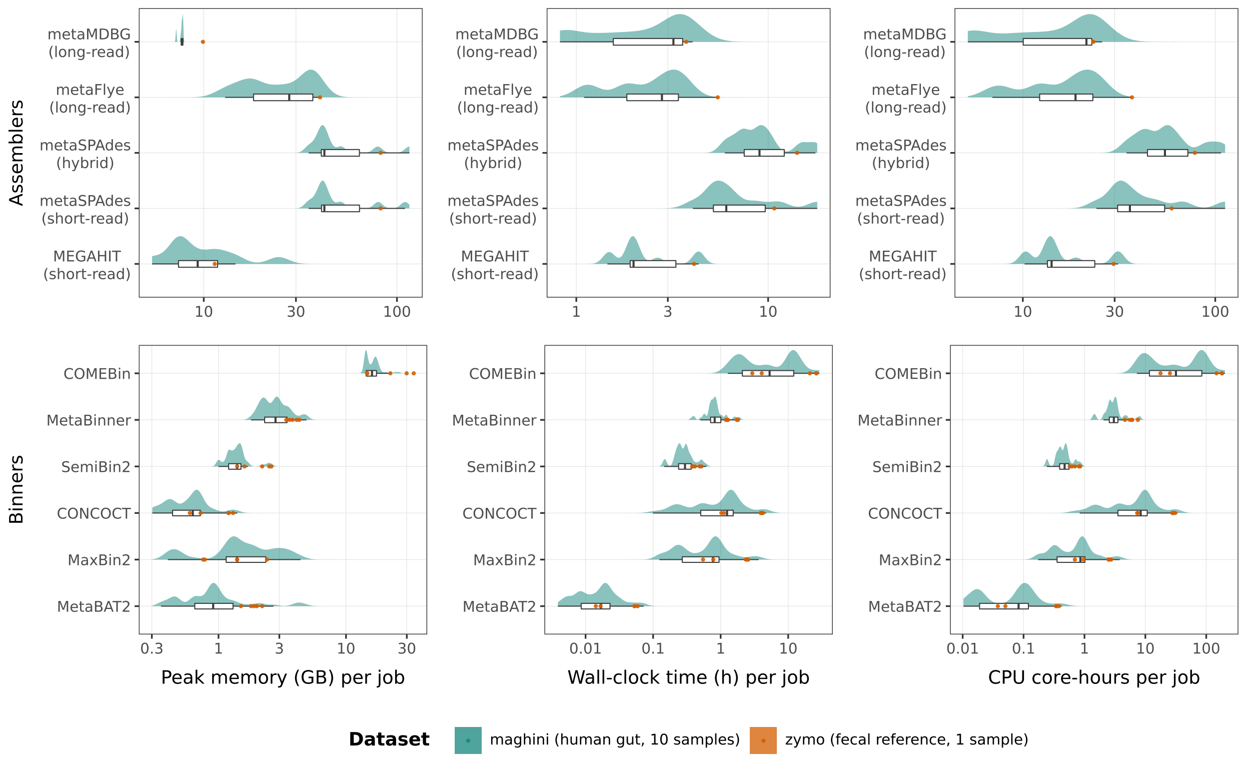

Supplementary Figure S4. Resource footprint per assembler and binner. Peak memory, wall-clock time, and CPU core-hours for assemblers (top) and binners (bottom). One kernel density scaled to a common height is shown for maghini at each tool, while zymo appears as points, since it has too few observations per tool. The box-plot in the middle pools both datasets.

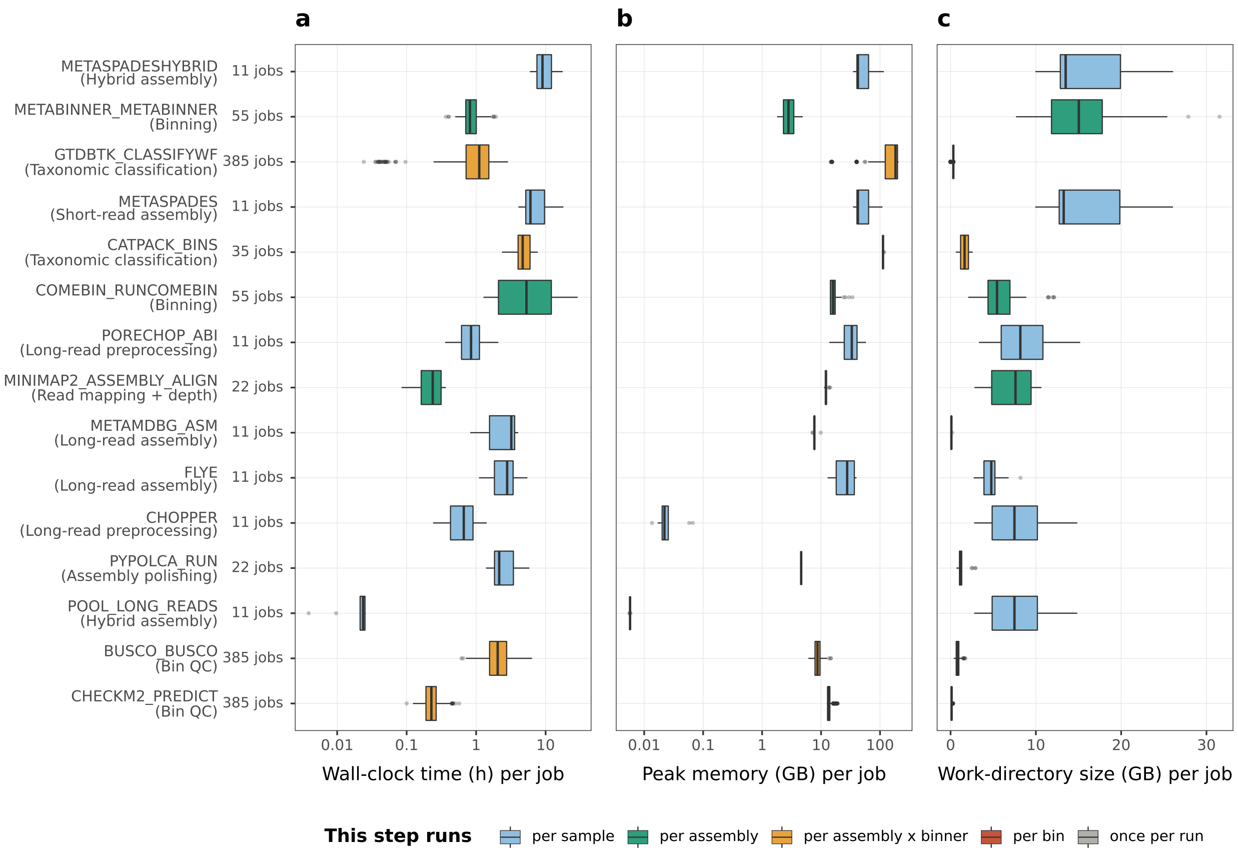

Supplementary Figure S5. Most resource-intensive processes per job. Processes with the highest median wall-clock time (a), peak memory (b), or work-directory size (c) per job. Each row is labelled with the process over the analysis stage it belongs to, followed by the number of jobs it ran. Each process is ranked separately by its median for each of the three resources, and processes are then ordered by their best (lowest) of those three ranks, with the sum of the three ranks breaking ties; the 15 highest-ranked are shown. Time and memory use logarithmic scales. The peak memory reported for GTDB-Tk (median 183 GB) overstates what it needs because its memory-mapped reference data is counted as resident in every job; it ran without failures on the 140 GB recommended by its developers.

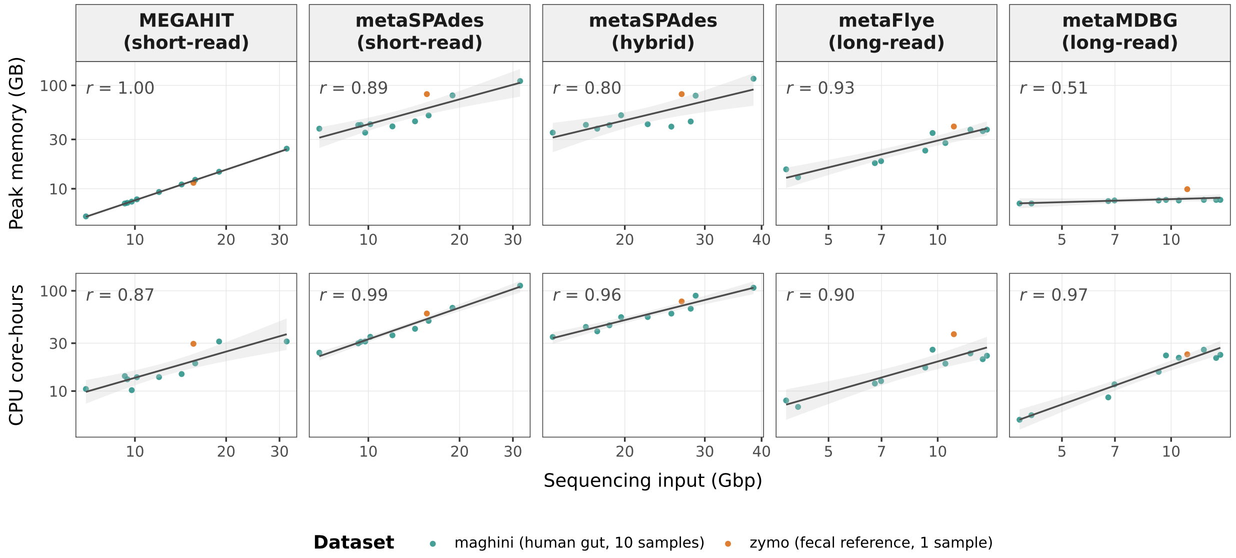

Supplementary Figure S6. Assembler scaling with sequencing input. Assembler peak memory (top) and CPU core-hours (bottom) as a function of sequencing input. Every assembler was allocated 8 CPU cores. Input corresponds to short reads, long reads, or their sum according to the assembler. Points represent samples, grey bands show linear fits on log–log scales. Each panel is annotated with r, the Pearson correlation coefficient between the log-transformed variables, pooling both datasets.

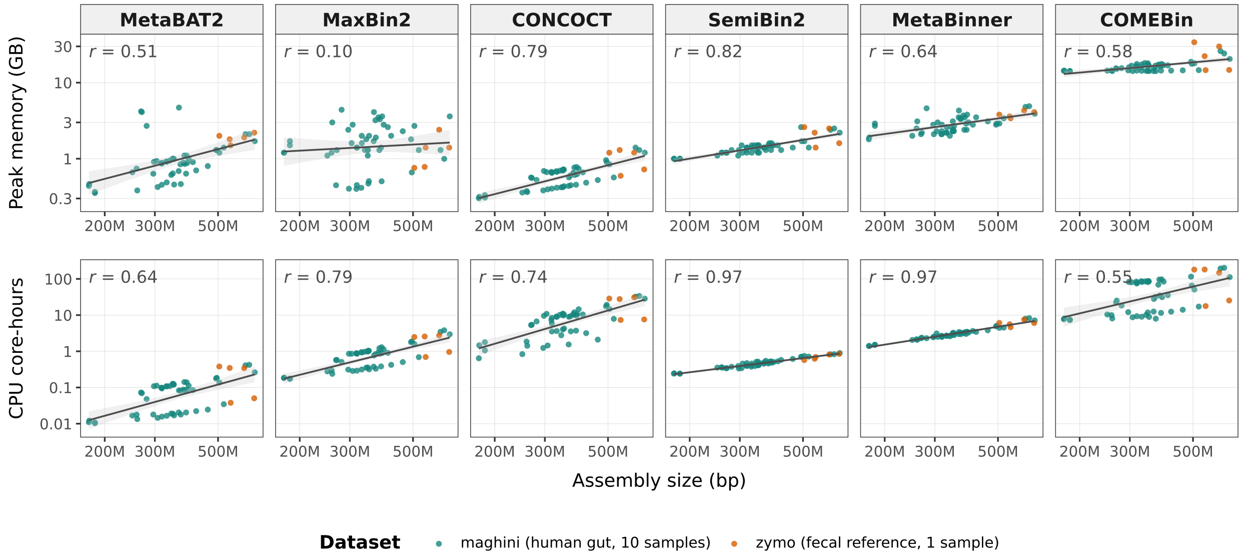

Supplementary Figure S7. Binner scaling with assembly size. Binner peak memory (top) and CPU core-hours (bottom) as a function of input assembly size. Every binner was allocated 8 CPU cores. Points represent assembly–binner combinations, grey bands show linear fits on log–log scales. Each panel is annotated with r, the Pearson correlation coefficient between the log-transformed variables, pooling both datasets.
